# Elafin is related to immune infiltration and could predict the poor prognosis in ovarian cancer

**DOI:** 10.3389/fendo.2023.1088944

**Published:** 2023-01-19

**Authors:** Weiyu Lu, Biao Xie, Guangqing Tan, Wanying Dai, Jingyi Ren, Sadaf Pervaz, Kun Li, Fangfang Li, Yingxiong Wang, Meijiao Wang

**Affiliations:** ^1^ Department of Physiology, School of Basic Medical Science, Chongqing Medical University, Chongqing, China; ^2^ Department of Biostatistics, School of Public Health and Management, Chongqing Medical University, Chongqing, China; ^3^ Joint International Research Laboratory of Reproduction and Development of the Ministry of Education of China, School of Public Health and Management, Chongqing Medical University, Chongqing, China

**Keywords:** ovarian cancer, elafin, immune infiltation, prognosis, CIBERSORT, nomogram, IHC analysis

## Abstract

**Background:**

Ovarian cancer (OC) is the most lethal gynecologic malignancy, yet the clinical results for OC patients are still variable. Therefore, we examined how elafin expression affects the patients’ prognoses and immunotherapy responses in OC, which may facilitate treatment selection and improve prognosis.

**Methods:**

The elafin mRNA expression profile was downloaded from The Cancer Genome Atlas (TCGA) and Gene Expression Omnibus. Elafin’s prognostic potential and its relationship with clinical variables were investigated using Kaplan–Meier survival curves, time-dependent receiver operating characteristic curves as well as univariate and multivariate Cox regression models. As validation, protein expression in the tumor and adjacent tissues of OC patients was investigated by using immunohistochemistry (IHC). Comprehensive analyses were then conducted to explore the correlation between immune infiltration and elafin expression.

**Results:**

A higher mRNA expression of elafin was associated with an unfavorable prognosis in TCGA cohort and was validated in GSE31245 and IHC. Moreover, elafin was indicated as an independent risk factor for OC. A significantly higher protein expression of elafin was detected in the adjacent tissues of OC patients with shorter overall survival (OS). The immune-related pathways were mainly enriched in the high-elafin-mRNA-expression group. However, the mRNA expression of elafin was favorably correlated with indicators of the immune filtration and immunotherapy response, which also proved better immunotherapy outcomes.

**Conclusion:**

The high elafin expression was associated with an unfavorable OS, while it also indicated better immunotherapy responses. Thus, the detection of elafin is beneficial to diagnosis and treatment selection.

## 1 Introduction

Ovarian cancer (OC) is known as one of the most lethal gynecologic malignancies, ranking as the fourth malignancy with the highest mortality rate (1). In 2020, approximately 313,959 women would have been newly diagnosed with OC, and 207,252 patients would have died as estimated. Compared with the regional concentration of other cancers, OC is prevalent across the world (2). The main obstacle to recovery and survival following therapy is thought to be the fact that the majority of patients have severe diseases upon initial diagnosis (1, 3). The National Comprehensive Cancer Network suggested comprehensive treatment plans for individuals with advanced-stage OC (stages III to IV), which include surgery, chemotherapy, and targeted therapy (4, 5). Targeted therapy has been incorporated into normal treatment routines in recent years. Bevacizumab was among the earliest to be used as targeted drugs (6). For platinum-sensitive OC, bevacizumab combined with carboplatin/gemcitabine significantly improved progression-free survival (PFS) and overall survival (OS) *versus* carboplatin/gemcitabine alone (7). Moreover, poly ADP-ribose polymerase (PARP) inhibitors have also been approved for use in OC and are recommended as the first-line maintenance therapy in patients with BRCA1/2 mutations and advanced disease, thus lowering the risk of disease progression or death. For patients with a recurrence, surgery integrated with the use of antiangiogenic agents and PARP inhibitors has a positive effect on their survival (5, 8, 9). With the development of immunotherapy for cancers, off-target effects have been considerably reduced by enhancing immune responses and protecting the patients against tumors (6). Immunotherapy, which also has cell toxicity and side effects, has emerged as a potent strategy for treating OC, while chemotherapy struggled with drug resistance and recurrence (10–13). The PD-1/PD-L1 pathways are the most common check targets of the immune system (14). Although it is validated that a combination of PD-1 antibodies (pembrolizumab and nivolumab) and PD-L1 antibodies (avelumab, atezolizumab, and durvalumab) enhances the efficiency of OC patients’ treatment (15, 16), the response rates of the patients are still relatively low (12, 17). Immune-related adverse effects undermine the effectiveness of immunotherapy, which also shortens the PFS and the OS (18). Therefore, to improve the effectiveness of immunotherapy, it is critically necessary to develop a mechanism that can accurately and reliably predict the patients’ immune response rates before treatment.

Elafin, also called peptidase inhibitor 3, is a protein-encoding gene located on chromosome 20q12-13 (19). In 1989, it was found to be related to epidermis inflammatory response (20, 21). As a member of the whey acidic protein four-disulfide core (WFDC) family, elafin is involved in the regulation of inflammation and protection against tissue damage as well as prevention of elastase-mediated tissue proteolysis (22). Apart from epidermis diseases, elafin is also associated with the reproductive system. Elafin concentrations and subcellular localization may be indicators of the severity of cervical cancer (23). There are conflicting reports concerning elafin’s role in OC progression. Previous studies demonstrated that the upregulation of elafin and elafin-positive cells was associated with poor outcomes in OC as well as lowered the sensitivity to cisplatin, a genotoxic chemotherapeutic agent (24–26). However, the expression of elafin in normal tissues was higher than that in ovarian tumor specimens (26). Herein it has not been certified yet whether elafin is a reliable marker for predicting the prognosis of OC patients.

In the present study, we conducted a comprehensive analysis, based on mRNA expression databases retrieved online, to explore whether elafin expression was correlated with OC prognosis and whether elafin was an independent prognostic marker. Enrichment analyses were employed to probe elafin-related pathophysiological mechanisms involved in the immune system, which were followed by a further analysis connecting the expression of elafin with immune infiltration and immunotherapy response. Combining the abovementioned analyses, we confirmed that elafin is a reliable biomarker of prognosis and immunotherapy response rates for OC patients.

## 2 Materials and methods

### 2.1 Workflow chart of the study

**Figure 1:**
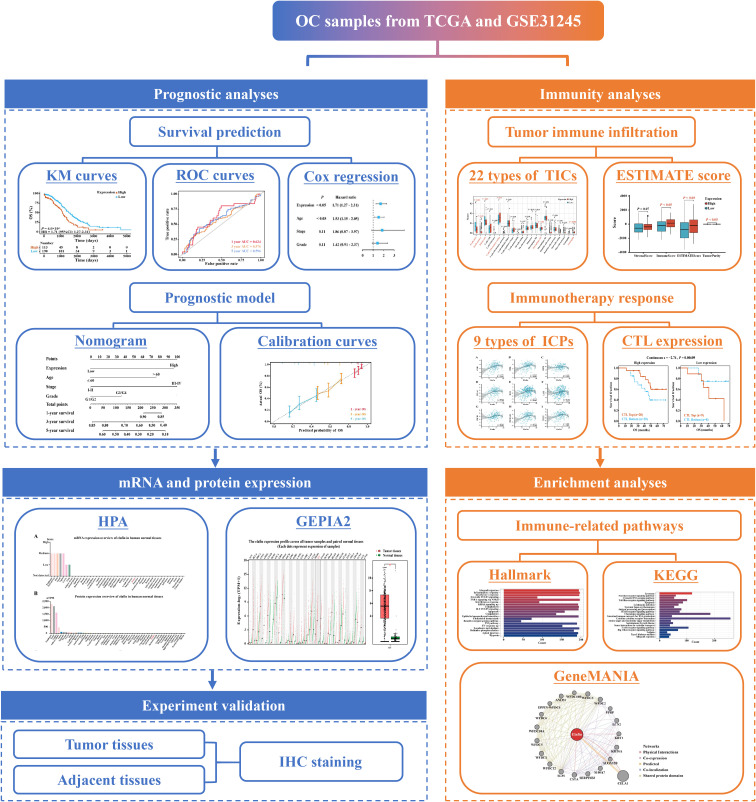
Workflow chart of this study.

The steps of our study were displayed in the flow chart (**Figure 1**), including data collection, grouping standards, general analyses, and a validation in an immunohistochemistry (IHC) experiment.

### 2.2 Data collection

We procured data, including the clinical information of 303 samples, from the University of California Santa Cruz (UCSC) website (https://xenabrowser.net/) and the mRNA expression of samples in GSE31245 from the Gene Expression Omnibus (GEO; https://www.ncbi.nlm.nih.gov/geo/). The cohort from UCSC was used as the training set, while the cohort of GSE31245 was employed as the verification set. Only samples with complete survival information and OS longer than 1 day could be included in our analyses.

### 2.3 Survival predicted by the expression of elafin and clinical factor

Depending on the cutoff expression of elafin, samples in The Cancer Genome Atlas (TCGA) were divided into high- and low-expression cohorts. To validate the prognostic value of elafin, we computed the correlation between gene expression and OS in OC by using the Kaplan–Meier (KM) survival curve. The time-dependent receiver operating characteristic (ROC) curve was a rule measuring the predictive capacity of elafin for OS. External validation in the GEO cohort was operated. Patients in GSE31245 were likewise assigned into two groups. We also carried out the KM survival and time-dependent ROC curve analyses. In the time-dependent ROC curves, the area under the curve (AUC) was utilized to testify the predictive accuracy of the KM survival analysis.

Univariate and multivariate Cox regression analyses were deployed to examine whether elafin was an independent and valuable risk factor for OC. It was also assessed whether the clinical characteristics (age, stage, and grade) of the patients affected their OS.

### 2.4 Construction and validation of the prognostic nomogram

Based on elafin expression and clinical characteristics (age, stage, and grade), we constructed a nomogram to predict the 1-, 3-, and 5-year OS of OC patients. It was calibration curve (27) and decision curve analysis (28) that validated the prognostic efficiency and the clinical applicability of the nomogram.

### 2.5 A different expression of elafin in cancer and normal tissues

The Human Protein Atlas (HPA, https://www.proteinatlas.org/) is an integrated tool to study the distribution of 17 protein-coding genes and their individual impacts on clinical outcomes in common human cancers. GEPIA2 (http://gepia2.cancer-pku.cn/), an enhanced version of GEPIA, is a comprehensive web server based on RNA sequencing data of tumors from TCGA and normal samples from Genotype–Tissue Expression (GTEx) cohorts (29, 30). We employed HPA to show the protein and mRNA expression of elafin. The “General” module on the web server GEPIA2 was also utilized to show the elafin expression across all tumor samples and paired normal tissues in a dot plot and box plot.

### 2.6 IHC analyses

To analyze the protein levels of elafin, tissue microarray (TMA) sections were purchased, including 45 OC samples paired with non-tumor ovarian tissues (six of 90 were invalid), from Superbiotek Pharmaceutical Technology (Shanghai, China). The TMA sections (4 μm in thickness) were baked overnight at 60°C, followed by 15 min of incubation with sodium citrate buffer (10 mmol/L, pH = 6.0) in the microwave, and then the slides were washed with graded phosphate-buffered saline. After blocking endogenous peroxidases and nonspecific antigens, the sections were firstly incubated with elafin polyclonal antibody (1:400, Proteintech, China) overnight at 4°C and secondly incubated with the secondary antibody (Zsbio, China) for 30 min at room temperature. After 2 min of reaction with diaminobenzidine and 5 min of counterstaining with hematoxylin, two professional pathologists independently assessed the IHC scores of the samples without access to the clinical information.

The staining scores of each sample were calculated by using the following formula: staining score = staining intensity × percentage of positive tumor cells × 100. The staining intensity was graded as follows: 0—no staining, colorless; 1—weak staining, light yellow; 2—moderate staining, yellow-brown; and 3—strong staining, brown. The tumor cell proportion was graded as follows: 0—no positive tumor cells, 1—< 10% positive tumor cells, 2—10–25% positive tumor cells, 3—26–49% positive tumor cells, and 4—≥ 50% positive tumor cells. The staining scores were defined as follows: 0, negative; 1–4, weakly positive; 5–8, positive; and 9–12, strongly positive.

### 2.7 Estimation of the immune infiltration and immunotherapy response

CIBERSORT, ESTIMATE, and TISIDB (http://cis.hku.hk/TISIDB) were utilized together to explore the condition of tumor immune infiltration. The “estimate” package (31) was utilized to calculate the ESTIMATE score, immune score, stromal score, and tumor purity, and their association with elafin expression was explored for samples from TCGA. The “CIBERSORT” package, a deconvolution algorithm based on RNA mixtures, was used to figure out the proportions of 22 tumor-infiltrating immune cells (TICs) coupled with LM22 (32). The TISIDB online database provides the association between elafin and immune features containing 28 types of TICs, immunomodulators, and chemokines (33). We downloaded the expression data of immune checkpoints (ICPs) to investigate their association with elafin expression, which could predict the immunotherapy response of patients. TIDE (http://tide.dfci.harvard.edu/) (34, 35) is a tool predicting the immunotherapy response based on two primary mechanisms of tumor immune evasion. Cytotoxic T lymphocytes (CTLs) expression indicates of better responses of immunotherapy. Furthermore, a higher TIDE prediction score indicates a higher potential of tumor immune evasion, thus exhibiting a lower rate of immune checkpoint blockade (ICB) response more directly.

For further understanding on how elafin affected the immunotherapy response rate, we made prediction of some drugs related to elafin on the GSCAlite, which facilitated to find associated drugs based on CDSC and CTRP databases (36).

### 2.8 Pathway enrichment analyses

Gene Set Enrichment Analysis (GSEA) was performed to explore biological signaling functions and pathways in the high- and low-expression groups (37, 38). HALLMARK and Kyoto Encyclopedia of Genes and Genomes (KEGG) pathways were ranked in ascending order of normalized enrichment score (NES). Biological functions with |NES| > 1, NOM *P*-value < 0.05, and false discovery rate *Q*-value <0.25 were significantly enriched. GeneMANIA (http://www.genemania.org/) was further used to build a biological network to explore elafin's functions and elafin-related genes, which assisted in confirming elafin’s role in functional pathways repeatedly (39). The correlation network was modified using Cytoscape software (v3.9.0) to better present the interrelationship.

### 2.9 Statistical analyses

Statistical analyses were performed with R software (v4.0.4). The “survival” R package (v3.2.7) and the “survminer” R package (v0.4.9) were used to plot the KM survival curves and to conduct the univariate and multivariate Cox regression analyses. The “survivalROC” R package (v1.0.3) was used to plot the time-dependent ROC curves. The “rms” R package (v6.2-0) was used to construct the nomogram and calibration curves. We used the Mann–Whitney *U*-test to analyze the difference in TIC ratios and ESTIMATE results between different elafin expression groups, and Student’s *t*-test for the association of TIDE scores with elafin expression. Spearman rank tests were performed for correlation analysis. A two-sided *P*-value < 0.05 was regarded as statistically significant. The “ggplot2” R package (v3.3.5) was employed for visualization.

## 3 Results

### 3.1 Upregulation of elafin was correlated with the poor prognosis for OC

**Figure 2:**
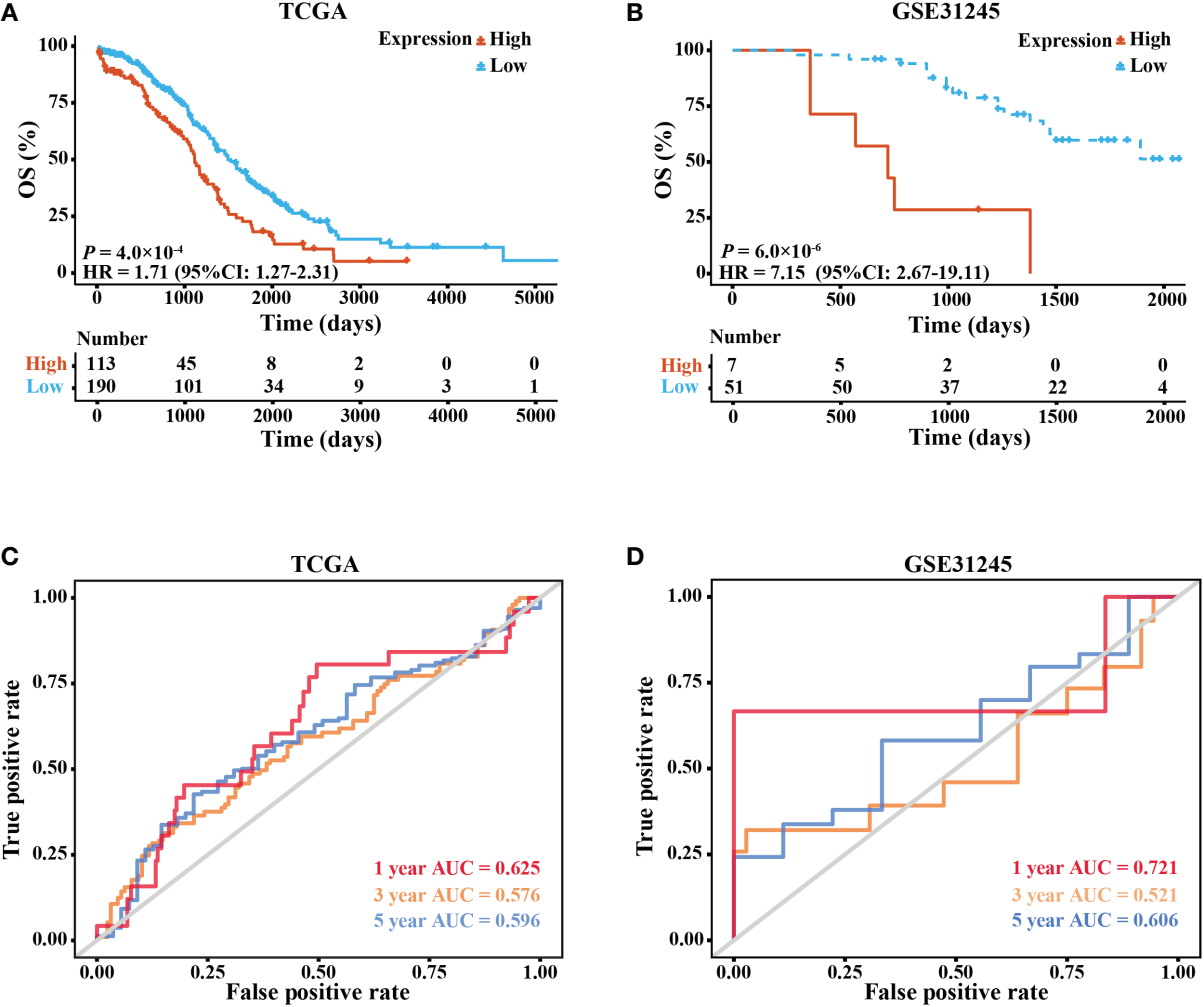
Expression of elafin and its correlation with the prognosis of ovarian cancer patients. **(A, B)** Five-year Kaplan–Meier survival curves comparing the high- and low-elafin-expression groups in The Cancer Genome Atlas (TCGA) and GSE31245. **(C, D)** Time-dependent receiver operating characteristic curves (1, 3, and 5 years) of elafin for predicting the patients’ overall survival in TCGA and GSE31245.

The KM survival curves based on data from TCGA showed that patients in the high-expression group had a shorter 5-year OS than those in the low-expression group (HR = 1.71, 95% CI: 1.27–2.31, *P* = 0.0004; **Figure 2A**). Then, correlation analyses between the patients’ OS and three clinical features (age, stage, and grade) were also carried out. Additionally, we also plotted the KM survival curves of the data from the GSE31245 cohort, which validated the poor prognosis of patients in the high-expression group (HR = 7.15, 95% CI: 2.67–19.11, *P* = 0.000006; **Figure 2B**).

As shown in **Supplementary Figures S1A–F**, the correlation of increased elafin expression with poor OS was also significant in age ≤60 (HR = 1.91, 95% CI: 1.27–2.87, *P* = 0.001), late-stage (HR = 1.80, 95% CI: 1.33–2.45, *P* = 0.0001), and late-grade groups (HR = 1.82, 95% CI: 1.32–2.50, *P* = 0.0002), respectively.

The ROC curves of TCGA cohort confidently demonstrated that elafin could predict the prognosis of OC (1-year AUC = 0.625, 3-year AUC = 0.576, and 5-year AUC = 0.596), which was likewise confirmed in the GSE31245 cohort (1-year AUC = 0.721, 3-year AUC = 0.521, and 5-year AUC = 0.606; **Figures 2C, D**).

### 3.2 Elafin was an independent risk factor for OC

**Figure 3:**
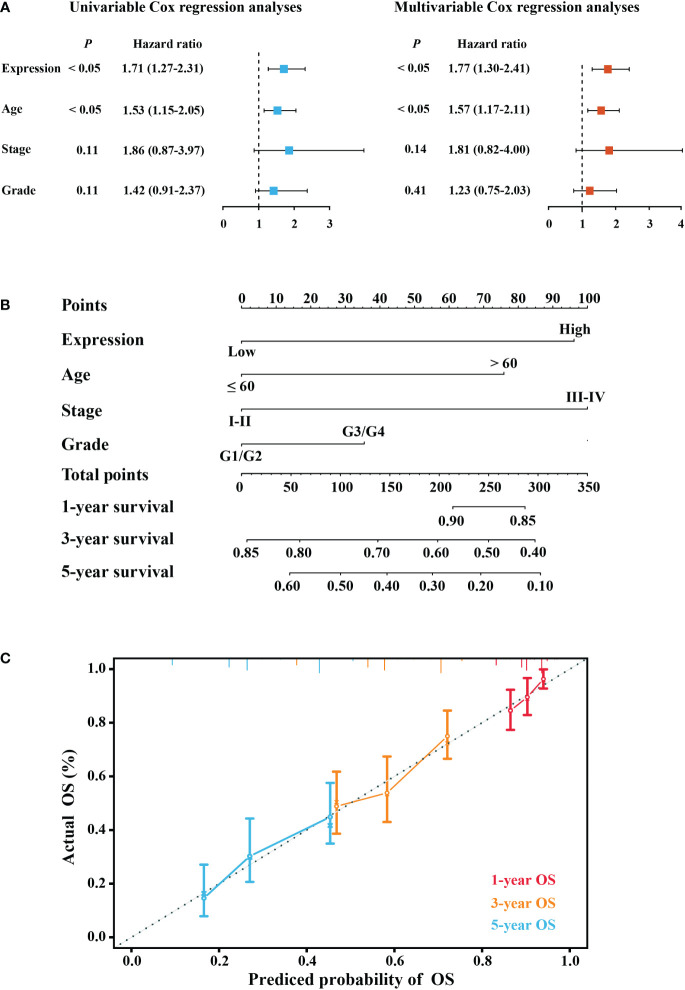
Construction of a predictive model. **(A)** Forest plot of univariable Cox and multivariable Cox regression analyses. **(B)** Nomogram model predicting the 1-, 3-, and 5-year overall survival (OS) combining elafin expression and clinical characteristics. **(C)** Calibration curves of 1-, 3-, and 5-year OS probability.

There was no correlation between the clinical characteristics of TCGA cohort and elafin mRNA expression as shown in **Table 1**. By conducting the univariate and multivariate Cox regression analyses in the TCGA cohort, it was clear that elafin was an independent and valuable risk factor for OC patients (**Figures 3A, B**). The univariate Cox regression analyses showed the relation between the expression of elafin and prognosis of patients (HR = 1.71, 95% CI: 1.27–2.31, *P* < 0.05). It was interesting that age (HR = 1.53, 95% CI: 1.15–2.05, *P* < 0.05) was another risk factor other than stage and grade. The results of the multivariate Cox regression analyses were corresponding with those in the former, and elafin expression level (HR = 1.77, 95% CI: 1.30–2.41, *P* < 0.05) and age (HR = 1.57, 95% CI: 1.17–2.11, *P* < 0.05) were considered as risk factors for OC.

**Table 1:** Clinical data of ovarian cancer samples from The Cancer Genome Atlas.

	Elafin mRNA expression		
	Tumor tissues (*n* = 303)		
	Low	High	*P*-value
Total	190 (62.71%)	113 (37.29%)	–
Age			
≤ 60	109 (57.37%)	65 (57.52%)	0.640
> 60	81 (42.63%)	48 (42.48%)	
TNM stage			
I/II	13 (6.84%)	9 (7.96%)	0.760
III/IV	177 (93.16%)	102 (90.27%)	
NA	0 (0%)	2 (1.77%)	
pN status			
N0	24 (12.63%)	20 (17.70%)	0.710
N1	51 (26.84%)	32 (28.32%)	
NA	115 (60.53%)	61 (53.98%)	
Histologic grade			
G1/G2	24 (12.63%)	10 (8.85%)	0.440
G3/G4	163 (85.79%)	98 (86.73%)	
NA	3 (1.58%)	5 (4.42%)	
Event			
Locoregional disease	3 (1.58%)	1 (0.89%)	–
Metastatic	1 (0.53%)	0 (0%)	
Progression of disease	9 (4.74%)	3 (2.65%)	
Recurrence	95 (50.00%)	48 (42.48%)	
NA	82 (43.15%)	61 (53.98%)	
Histological subtype			
Serous	190 (100%)	113 (100%)	–
Tumor residual			
> 20 mm	24 (12.63%)	28 (24.78%)	0.28
11–20 mm	17 (8.95%)	6 (5.31%)	
1–10 mm	88 (46.31%)	46 (40.71%)	
No macroscopic disease	41 (21.58%)	17 (15.04%)	
NA	20 (10.53%)	16 (14.16%)	

Depending on the results of the multivariate Cox regression, we constructed a nomogram for better prediction of the 1-, 3-, and 5-year survival possibility of OC patients. With higher total points related to poorer OS, **Figure 3C** depicts that a high expression of elafin made the most notable contribution to an unfavorable prognosis. The calibration curves for 1-, 3-, and 5-year survival were plotted, and the prediction lines were closed to the ideal line, which validated the prognostic efficiency and the clinical applicability of the nomogram.

### 3.3 Elafin expression varied in tumor and normal tissues

**Figure 4:**
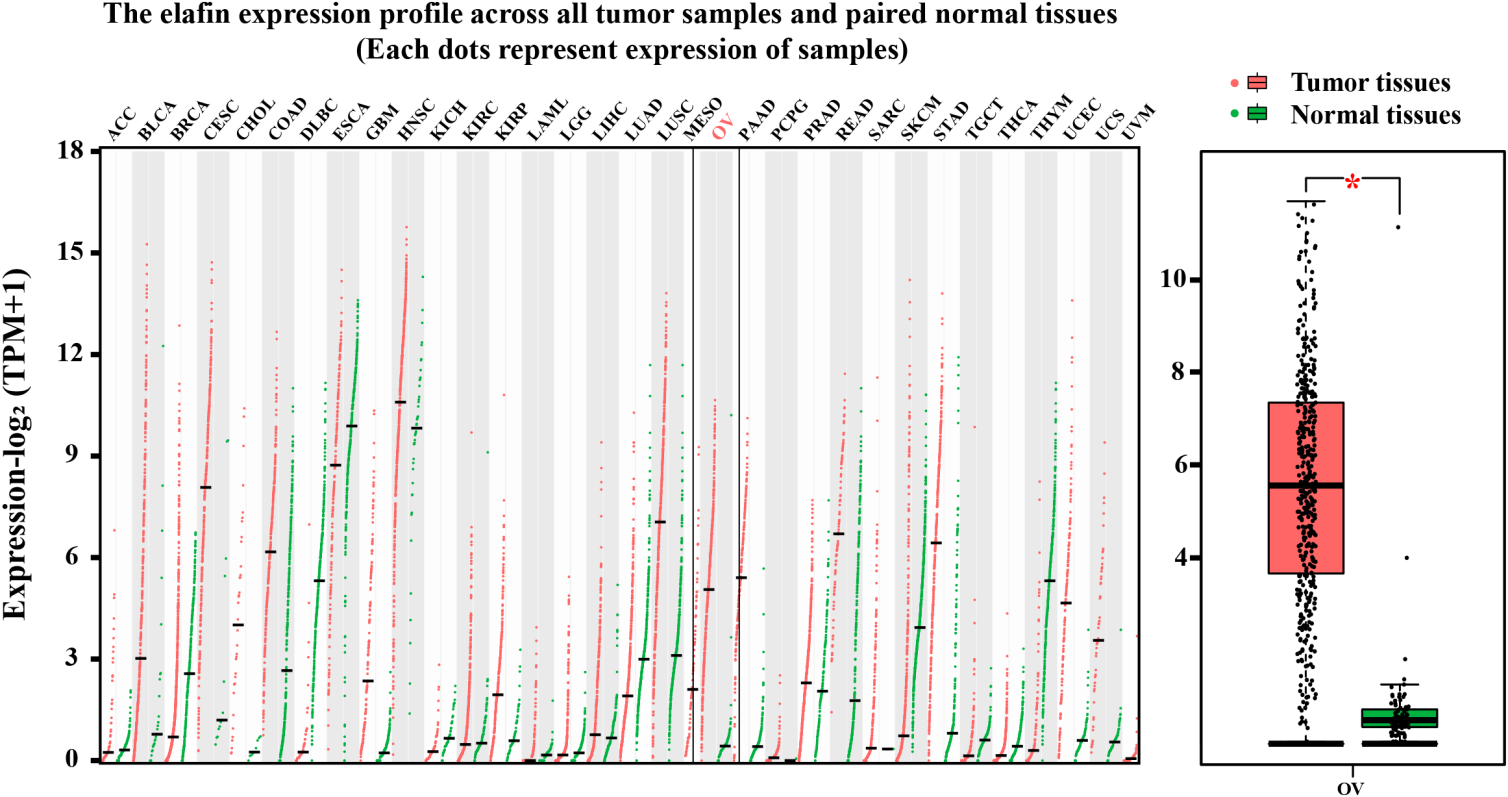
Results from GEPIA2 database. mRNA expression levels of elafin in different tumor tissues and corresponding normal organ tissues, and mRNA expression levels of elafin in ovarian cancer and normal ovary.

Generally, the protein expression and the mRNA level of elafin in normal and tumor tissues were relatively lower compared with other gene-related expressions. The mRNA average nTPM of the ovary and the median FPKM of OC were 0.7 and 23, respectively; even the protein of elafin was not detected in the ovary and OC (**Supplementary Figures S2A–D**). We conducted a further exploration on GEPIA2 and utilized visual plots to show that elafin expression varied from tumor to normal tissues in **Figure 4**. The expression level of elafin in OC (426 samples from TCGA) was much higher than that in normal tissues (88 samples from GTEx), and the difference was significant (*P* < 0.05).

### 3.4 IHC validation of elafin expression in OC tissues

**Figure 5:**
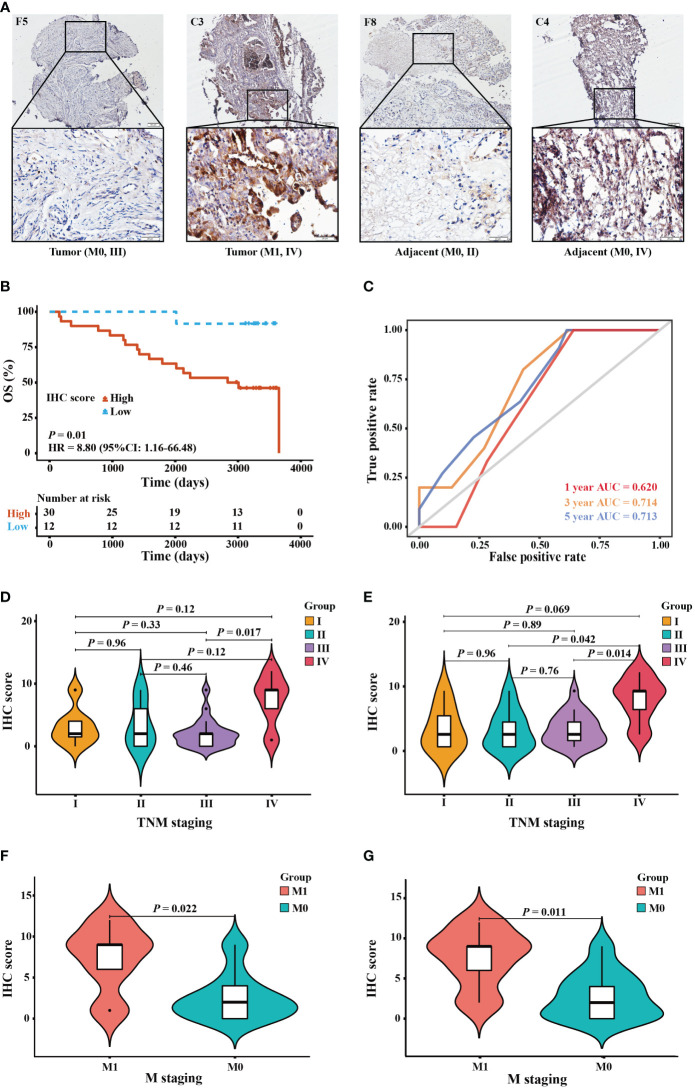
Protein expression and prognostic value of elafin in ovarian cancer patients by immunohistochemistry (IHC) staining validation. **(A)** IHC staining of elafin in tumor and adjacent tissues at different TMN stages. The images are presented at ×10 magnification (scale bar, 200 μm) and ×40 magnification (scale bar, 50 μm) *via* a microscope. **(B)** The Kaplan–Meier survival curve was plotted based on 42 tissue microarray adjacent specimens comparing the high- and low-score groups in terms of elafin expression. **(C)** The 1-, 3-, and 5-year time-dependent receiver operating characteristic curves of elafin for predicting the patients’ overall survival in adjacent tissues. **(D, E)** Difference of the IHC scores of different TMN stages in tumor and adjacent tissues, respectively. **(F)** Association of IHC scores in tumor tissues with cancer metastasis. **(G)** Association of IHC scores in adjacent tissues with cancer metastasis.

IHC staining was utilized to verify the bioinformatics results. In **Supplementary Table S1**, the clinical characteristics of patients and their associations with elafin protein expression in OC tissues and adjacent tissues are described. The elafin protein expression, both in tumor and adjacent tissues, had some connections with clinical characteristics. The late TMN stages had a higher elafin expression than the early stages, and metastasis of OC was often correlated with a high elafin protein expression detected in tumor tissues (*P* = 0.045; *P* = 0.022). Meanwhile, in the adjacent tissues, the high expression indicated a bigger primary tumor (*P* = 0.038) and more chances of metastasis and recurrence (*P* = 0.041; *P* = 0.026). The staining patterns of elafin in OC tissues and adjacent normal ovarian tissues are presented in **Figure 5A**. The KM survival curves based on the IHC scores of adjacent tissues showed that patients in the high-score group had a shorter 5-year OS than those in the low-score group (HR = 8.80, 95% CI: 1.16–66.48, *P* = 0.01; **Figure 5B**). The ROC curves demonstrated that elafin expression in adjacent tissues could predict the prognosis of OC patients (1-year AUC = 0.620, 3-year AUC = 0.714, and 5-year AUC = 0.713; **Figure 5C**). The IHC scores of elafin varied in different tumor node metastasis (TMN) staging in both tumor and adjacent tissues. In tumor tissues, the difference in IHC scores at stages III and IV was significant (**Figure 5D**). In adjacent tissues, the IHC scores at stage IV were significantly higher than those at stages II and III, respectively (**Figure 5E**). Since elafin protein can be secreted by OC cells, we therefore linked the IHC scores of elafin with OC metastasis. As **Figures 5F, G** show, OC patients with higher IHC scores in tumor or adjacent tissues were more likely to metastasize.

### 3.5 Tumor immune cell infiltration and its correlation with elafin expression

**Figure 6:**
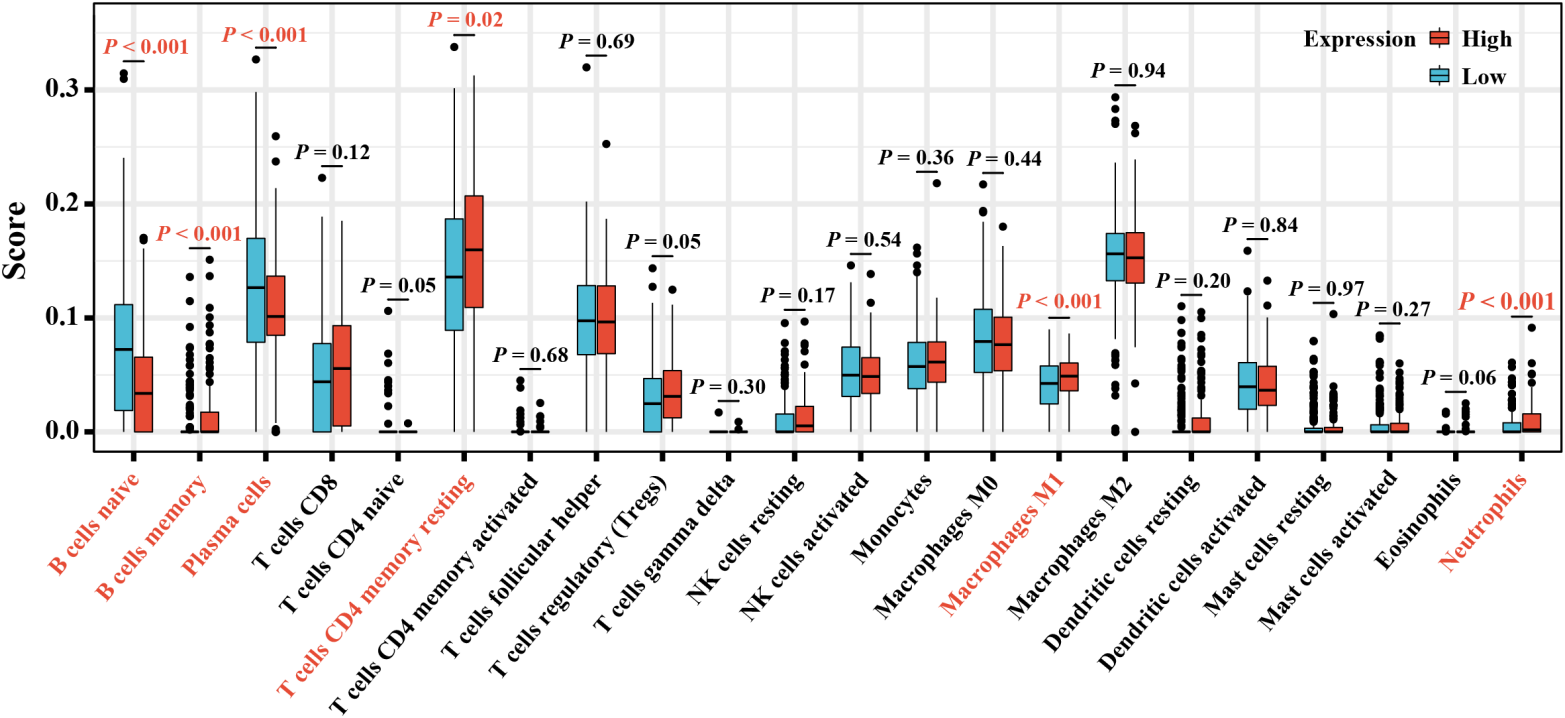
The box plot displays different proportions of the 22 types of TICs in the high- and low-expression groups.

A further exploration for the proportion of TICs in tumor tissues and their relationship with elafin expression was conducted using the CIBERSORT algorithm. While macrophage M2 accounted for most part in TIC infiltration (**Supplementary Figures S3A, B**), there were six types of TICs related to the expression of elafin, including B cells naive, B cells memory, plasma cells, T cells CD4 naive, macrophage M1, and neutrophils. Apart from B cells naive and plasma cells which were negatively associated with elafin expression, the proportion of the rest of the TICs mentioned was positively correlated with elafin expression (**Figure 6**).

ESTIMATE algorithm also helped to calculate immune infiltration. As indicated in **Supplementary Figure S4A**, elafin expression was positively related to immune scores and ESTIMATE scores (*P* < 0.05) and negatively related to tumor purity (*P* < 0.05). The heat map displayed in **Supplementary Figure S4B** shows the scores for each patient in TCGA with a continuous change in color.

### 3.6 Upregulation of elafin had the capacity to predict the immunotherapy response

**Figure 7:**
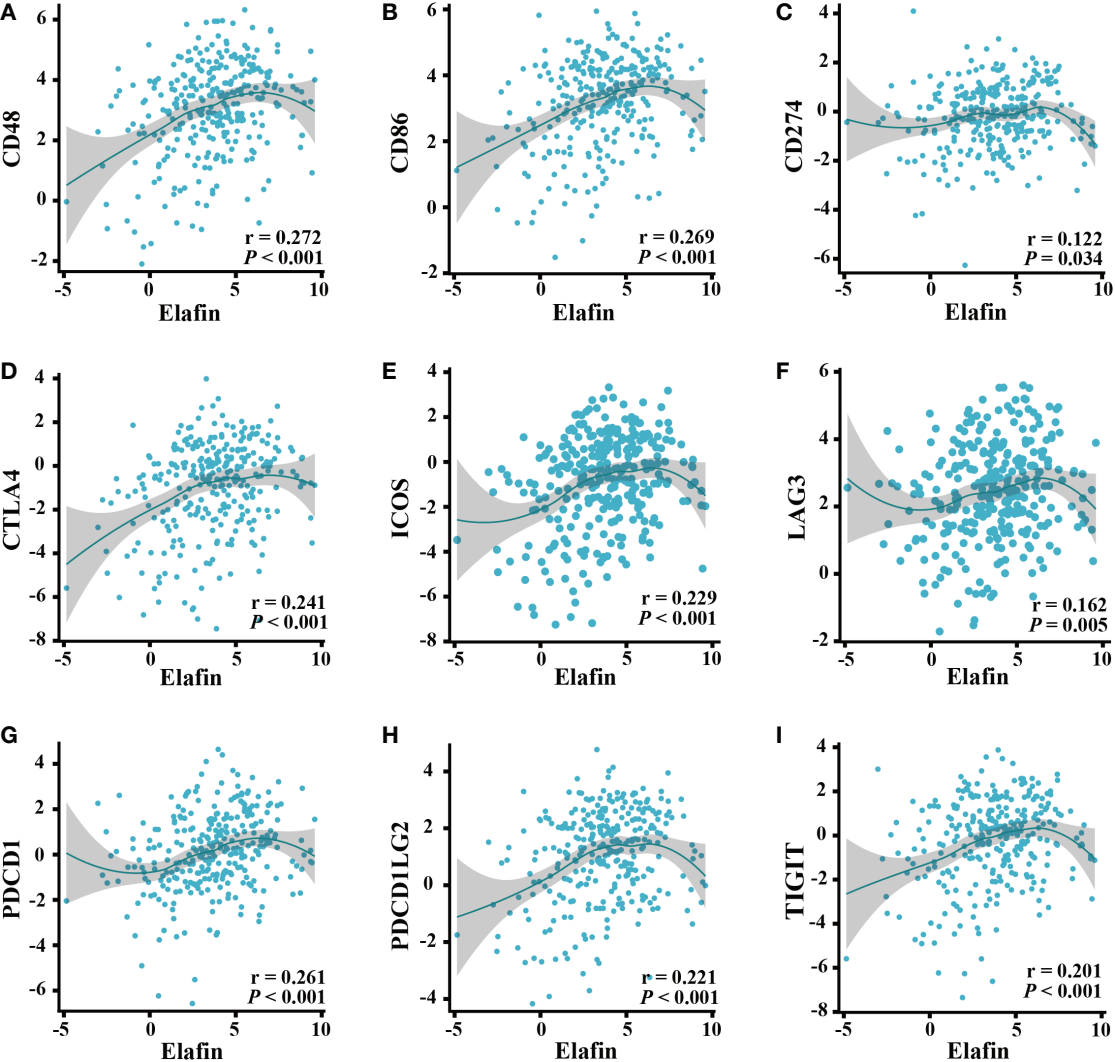
Correlation of ICPs and elafin expression in ovarian cancer patients. **(A)** CD48. **(B)** CD86. **(C)** CD274. **(D)** CTLA4. **(E)** ICOS. **(F)** LAG3. **(G)** PDCD1. **(H)** PDCD1LG2. **(I)** TIGIT.

The Spearman correlation analysis of data from the TISIDB web showed that the common ICPs (40), including PDL1 (CD274), CTLA4, ICOS, LAG3, PDCD1, CD48, CD86, and TIGIT, were all significantly associated with elafin expression (*P* < 0.05; **Figures 7A–I**).

**Figure 8:**
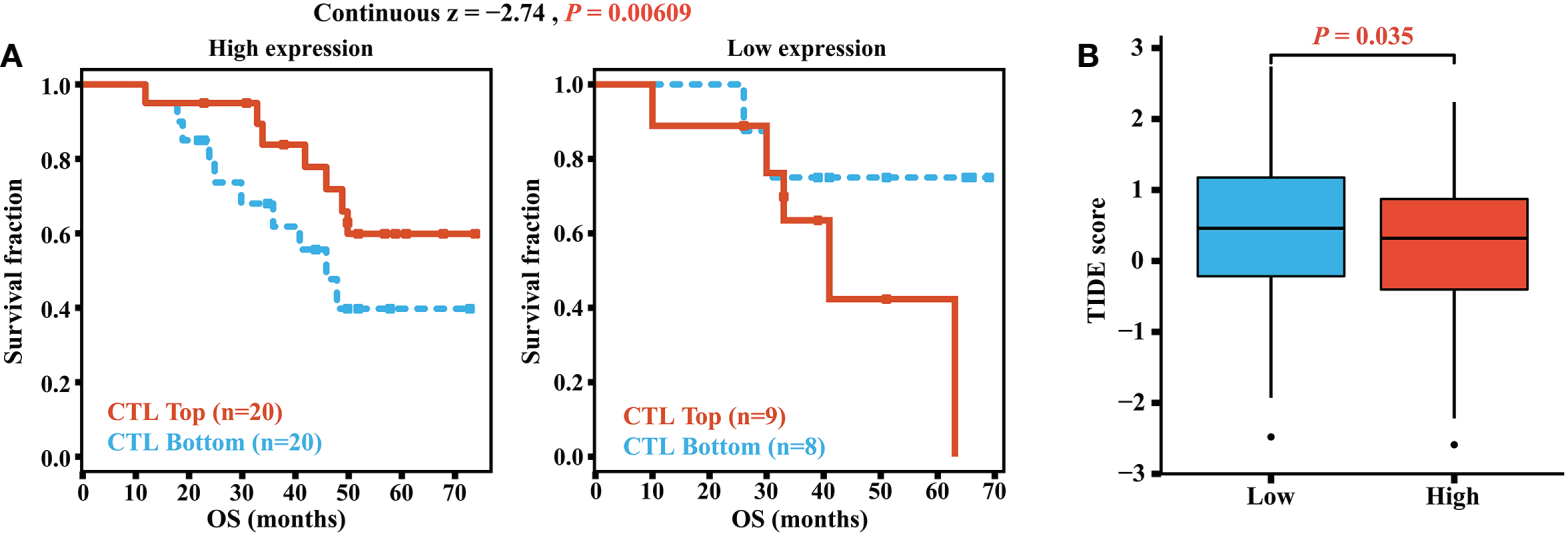
**(A)** Correlation of the cytotoxic T lymphocyte level and overall survival in groups with different levels of elafin expression. **(B)** TIDE scores in different high- and low-expression groups.

A higher infiltration of CTLs indicated better immunotherapy prognoses in OC patients. The GSE31245 cohort was included in the analyses of the TIDE online tools, and we found a difference in the prognostic value of CTLs. For OC patients with high elafin expression, high CTLs levels exhibited longer survival and better immunotherapy response, whereas we did not find this correlation in the low-elafin-expression group, which indicated the role of elafin (continuous *z* = -2.74, *P* = 0.00609, **Figure 8A**). In addition, we also utilized the server to determine the TIDE score depending on mRNA expression from the combination of TCGA and GSE31245. A higher TIDE score is positively related to greater potential of tumor immune evasion, which indicates unfavorable immunotherapy outcomes for patients. As shown in **Figure 8B**, the score was much higher in the low-expression group than that in the high-expression group (*P* = 0.035), which means that a higher elafin expression was correlated with better immunotherapy responses. Overall, the above-mentioned results suggested that elafin may play an important part in predicting the efficiency of immunotherapy.

The drug sensitivity and the mRNA expression profile data from GSCAlite were collated to understand the role of elafin in different therapies. Because few immunotherapy drugs were detected, it could not be determined if elafin was indeed helpful for better immunotherapy in terms of drug prediction. However, it was certified that elafin was sensitive to targeted drugs and resistant to chemotherapy drugs (**Supplementary Table S2**).

### 3.7 Elafin was associated with immunity pathways in OC

**Figure 9:**
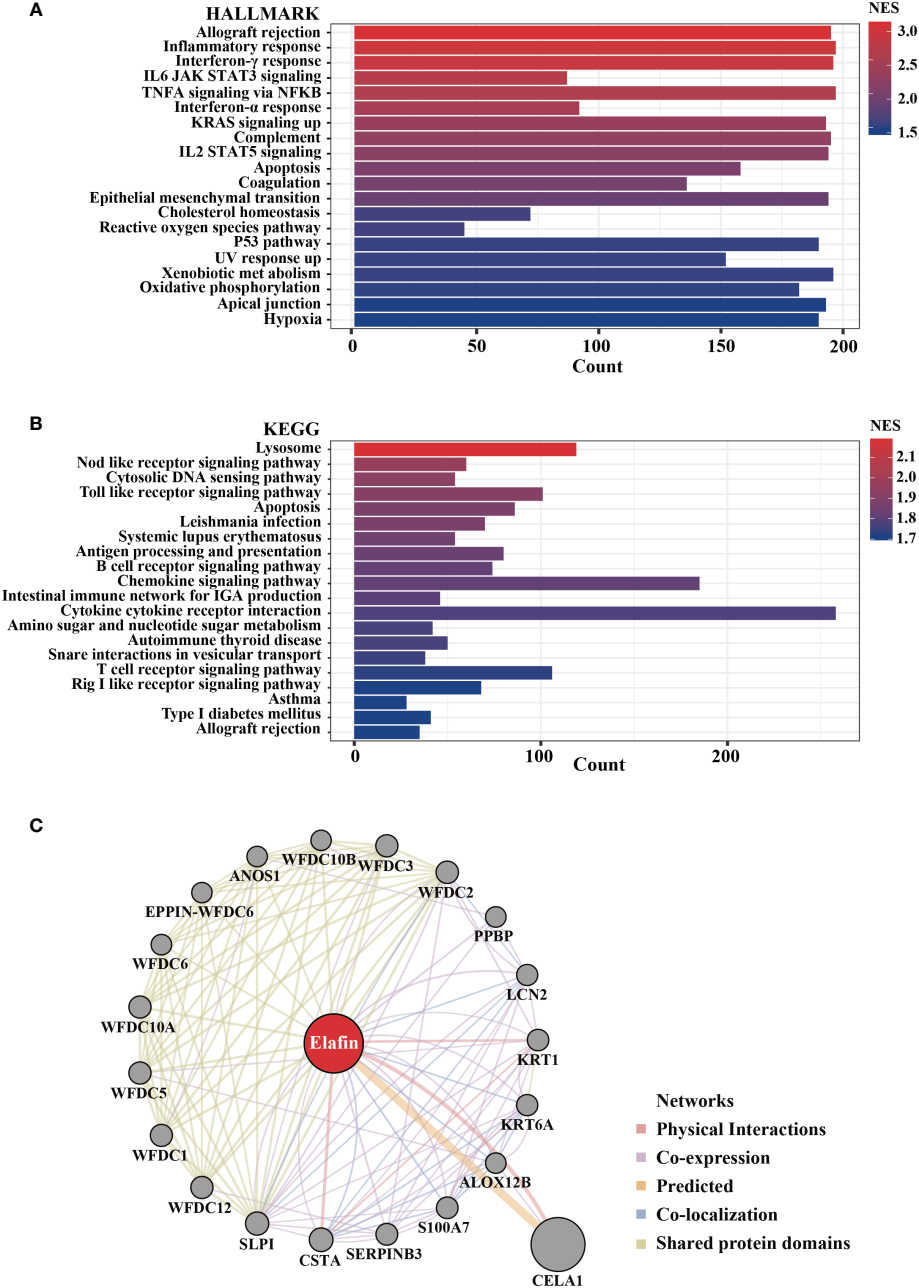
Biological pathways enriched in high-elafin-expression group as determined by Gene Set Enrichment Analysis based on The Cancer Genome Atlas cohort. **(A, B)** Top 20 function enrichment pathways in HALLMARK gene sets and Kyoto Encyclopedia of Genes and Genomes pathway; only gene sets with |NES| > 1, NOM *P <* 0.05, and false discovery rate *Q <* 0.25 were considered significantly enriched. **(C)** Protein–protein interaction analysis of elafin from GeneMANIA software.

A high expression of elafin was related to the poor prognosis of OC; thus, we performed GSEA and GeneMANIA to clarify the biological processes participated in by elafin. Hallmark gene sets and the KEGG subset of canonical pathways were taken into consideration when we conducted GSEA. Functional pathways enriched in terms of the high-expression group are listed in **Supplementary Table S3**, and the top 20 pathways enriched in Hallmark and KEGG gene sets are plotted, respectively, as shown in **Figures 9A, B**.

In the analyses based on Hallmark gene sets, a high expression of elafin was widely related to immune reaction, like allograft rejection, inflammatory response, interferon-γ response, interferon-α response, IL6 JAK-STAT3 signaling, and so on. Elafin was likewise associated with the NOD-like receptor signaling pathway, Toll-like receptor signaling pathway, apoptosis, and B cell receptor signaling pathway based on KEGG pathways. As the GeneMANIA indicates in **Figure 9C**, elafin was likewise related to immune-related functions like humoral immune response and antimicrobial humoral response, which were consistent with the enrichment findings from GSEA. We also accessed and exhibited the protein–protein interaction of elafin, and the results covered 20 elafin-related proteins.

## 4 Discussion

Immunotherapy entered a new era after James Allison and Tasuku Honjo discovered the PD-1 inhibitor’s role in cancer treatment. There are three stages in the tumor process: elimination, equilibrium, and escape (41). The key points of immunotherapy are figuring out the specific mechanism of tumor escape across variable malignancies and then applying the most appropriate treatment methods (42). The therapeutic effects for OC patients are limited. The resistance of PARP inhibitors was due to the lack of homology-directed DNA repair (43), and the resistance of PD1/PD-L1 is because of T cell dysfunction, antigen recognition disorders, T cell activation disorders, *etc.* Immune infiltration in the tumor microenvironment is a prerequisite for immunotherapy, which is affected by the variable immune cell and chemokine proportions (44). Thus, the underlying mechanism of the interrelationship between immune infiltration and immunotherapy needs further exploration, which would not only help to understand the mechanism of drug resistance but also find out the involvement of a key signal for predicting immunotherapeutic efficacy.

A trio of WFDC genes, consisting of HE4, SLPI, and elafin, is certified to play roles in the aggressiveness of OC (45). Among the mentioned WFDC genes, HE4 shortens the survival time by altering the tumor immune microenvironment (46), and researchers have found a linear correlation between HE4 level and the peripheral monocyte/lymphocyte ratio, which could predict the survival of OC patients (47). SLPI could silence the neutrophil elastase’s anti-tumor effects, which are immune-related capabilities of attenuating tumorigenesis and attacking distant metastasis (48). Considering the same chromosome location and similarities with HE4 as well as SLPI, elafin’s role in ovarian tumorigenesis should be connected tightly with immune infiltration. Elafin was known as a risk factor for psoriasis arthritis and located in the epidermis (21) as reported in the last century. In recent studies, researchers have found its tight connection with reproductive system cancers such as OC (25), breast cancer (49), and cervical cancer (23). Previous studies have already found the correlation between elafin upregulation and an unfavorable prognosis of OC patients, but the particular reason why the relevance of elafin expression and OS varies in different stages of tumorigenesis is not clear enough (24, 50). Moreover, elafin-related immune infiltration requires more attention. Thus, we designed and conducted a comprehensive analysis of elafin to explore how it affected the OS at different stages of tumor development and the immunotherapy sensitivity of patients. Our research found the underlying mechanism and clarified the clinical values of elafin.

We downloaded information about OC patients from online databases, with TCGA database used as the training cohort and GSE31245 used as the verification cohort. The KM survival analyses showed that a high expression of elafin was related to an unfavorable prognosis. The univariate and multivariate Cox regression analyses certified that elafin was an independent risk factor (51) for OC. Moreover, the ROC curve and nomogram constructed an elafin-centered model to predict the 1- 3-, and 5-year OS for patients. Interestingly, there were no significant differences between the high- and low-elafin-expression groups in the early stages and grades. The IHC results confirmed the bioinformatics prediction. Elafin protein is secreted into the blood after its coding (45). We found that the higher IHC scores in adjacent tissues were associated with a shorter OS. In adjacent tissues, the IHC scores at stage IV were significantly higher than those at stages II and III. Thus, the protein expression of elafin was more directive in the late stages. Elevated serum levels of elafin are associated with a bigger possibility of cancer metastasis, and the result of the difference analysis between the high- and low-score groups certifies it. Herein the protein expression of elafin detected in the blood could predict the metastasis of OC, which could be a reliable detection indicator, as a supplement to HE4 and CA125 especially in late TMN staging.

The immune scores and ESTIMATE scores were significantly higher in the high-expression group, which were correlated with a higher proportion of B cell, T cell CD4, neutrophil, and macrophage (52). On the contrary, higher tumor purity was displayed in low-expression OC samples. Tumor purity is significantly correlated with the clinical features, genomic expression, and biological characteristics of tumor patients (53). These non-cancerous components influence tumor growth, invasion, and metastasis (54), like infiltrating T-lymphocytes playing their antitumor roles in OC (51, 55). Therefore, the positive relation between elafin and non-cancerous components is correlated with a favorable prognosis.

As reported, the composition of TICs impacted the survival of tumor patients (56), and related treatments were put into use (57) by participating in the progression, recurrence, and invasion of tumors (58). Elafin expression was positively correlated with B cell memory, T cell CD4 naive, macrophage M1, and neutrophils as our study has revealed. Macrophage M1 assisted in harming tumor cells by releasing reactive oxygen species, nitrogen intermediates, and inflammatory cytokines (59) as well as improving the anti-tumor effects of ICB, both of which indicated that macrophage M1 could be a novel target for immunotherapy (59, 60). B cell memory was also significant in the immune response after ICB therapy. The underlying mechanism was that its existence promoted T cell response and the function of secreting cytokines (including IL-6 and interferon-γ) (61). A high expression of elafin should indicate prognosis-favorable immune infiltration and longer OS, but it was related to the poor prognosis at advanced stages and grades. Elafin is secreted by OC and circulates in the blood (45). As found by Joseph A Caruso et al. (26), the expression of elafin is on the decrease during ovarian tumorigenesis; the anti-tumor effects from immune infiltration could be inferior to invasiveness malignancy. Herein it is not contradictory that residual elafin-positive cells were correlated with poor prognosis while it could inhibit tumor progression, which was likewise certified in breast cancer (49). It is worth adding that the expression alteration is the reason why the mRNA and protein expression of elafin was relatively low in OC and normal tissues. In terms of elafin expression related to T cell exhaustion signatures and the prognostic capacity of CTLs, there ought to be potential for it to predict ICB responses (62, 63). In the high-expression group, the expression of ICPs was significantly upregulated and responded well to the immunotherapy. The TIDE scores certified the better immune responses in the high-elafin-expression group, too. Overall, elafin was certified as the indicator of better immunotherapy responses.

We tried to predict drugs that will certify elafin’s role in making the immunotherapy response rate better. The predicted immunotherapy drugs were few, so whether elafin was sensitive or resistant to them was uncertain. However, it was found that elafin was, surprisingly, related to high sensibility of targeted drugs like afatinib, gefitinib, cetuximab, erlotinib, lapatinib, and so on. Gefitinib, one of the epidermal growth factor receptor (EGFR)-tyrosine kinase inhibitors, inhibited the phosphorylation of EGFR in epithelial OC tumor cells (64). The clinical use of cetuximab is more flexible and has been studied extensively in OC. Cetuximab is also an EGFR inhibitor, and its combination with carboplatin plays a role in screened patients who were EGFR-positive and had relapsed platinum-sensitive OC (65). Drug resistance is common in chemotherapy, but with targeted therapy as adjunct it could be reduced. Methotrexate, docetaxel, and camptothecin are common chemotherapeutic drugs (66–68). In our predictive analysis, elafin is resistant to methotrexate and camptothecin, as a previous study showed that elafin decreases the sensitivity of human epithelial OC cells to several genotoxic agents (25), which may have an important implication in predicting the response of patients with epithelial OC to chemotherapy.

Enrichment analyses by GSEA and GeneMANIA indicated that a high expression of elafin was confirmed to be correlated with immune- and inflammation-related biological function, which affected the development of cancer. Independent of ICB, interferon-related cytokine-induced senescence is capable of arresting tumor cells (69). The interferon-γ response is involved in the innate and adaptive immune system. Predominantly produced by innate immune cells (70), it plays a crucial role in activating effector immune cells and antigen presentation to suppress tumor growth. Besides its capability of inducing macrophage differentiation, it also aids in the antitumor process (71). Thus, interferon-γ is dramatically important for predicting treatment success (72, 73). Interferon-α also belongs to the interferon family but plays roles in ICB, which enhances the effectiveness of anti-PD-1 in hepatocellular carcinoma (74). Apart from interferon-γ and interferon-α responses, the IL-6/JAK/STAT3 signaling pathway suppresses the anti-tumor immune response (75), the NOD-like receptor signaling pathway affects the stages of inflammation-associated tumorigenesis (76), and the Toll-like receptor signaling pathway was a crucial access to promote invasion in OC patients (77). As previous studies and GSEA results have shown, elafin is correlated with interferon-γ and lipopolysaccharide, which increases HLA/MHC I expression for the improved recognition by CD8+ T cells as an important component towards enhanced tumor antigenicity (78). How elafin participates in the immune-related pathways would be attributed to its location and domains. Elafin shares the most similarities with SLPI among the WFDC family (79). SLPI contains two WFDC domains, whereas elafin has a single WFDC domain and a transglutaminase substrate-binding domain (TSBD) (80). The WFDC domain is known to impart antiprotease activity to the molecule, which could provide broad anti-infective cover against those pathogens and less inflammation. SLPI spontaneously increases with antileukoproteinase 1 in patients with OC, where the WFDC domain plays a crucial role (80, 81). Antileukoproteinase 1 increases the expression of cyclin D1 gene, decreases the tumor-suppressor gene lysyl oxidase, and protects progranulin, a highly expressed factor in aggressive cancers, from proteinase degradation (82). Having similar OC-related characteristics with SLPI, TSBD facilitates elafin to participate in different mechanisms. Transglutaminase is the medium of TSBD combining with extracellular matrix proteins (80, 83). It is induced by many inflammatory cytokines like TGF-β, TNF-α, IL-1, and IL-6, influencing inflammation in cancer, by which the desmoplastic response of tumors involves an interplay between the invading tumor cells and the altered extracellular matrix (84). Although the direct mechanisms by which elafin regulates the immune system are not very clear, the role of elafin in OC inflammation includes sharing methods of interaction mode and key molecules with that in immune-related functions, which need to be given additional consideration. To summarize, elafin plays intricate roles in the progression and treatment of cancers. On one hand, elafin was related to an unfavorable prognosis of OC patients which involved apoptosis of tumor cells and the IL-6/JAK/STAT3 signaling pathway; on the other hand, a high expression of elafin participated in immunotherapy for tumors, indicating an ideal survival situation. The results from GeneMANIA were proof of GSEA and further provided elafin-related genes.

Nevertheless, there exist limitations to our present study. Firstly, we downloaded information about patients from a public database. The sample size was relatively small after filtering and sorting, which might have the risk of bringing bias to the results. To minimize the negative effects of limited samples, we confirmed our results by using online tools and IHC scores. Secondly, the detailed patients’ clinical information was not clear enough, and whether patients once accessed immunotherapy or chemotherapy affected the accuracy of the analyses. In clinical treatment, immunotherapy was often utilized in conjunction with chemotherapy. In our future studies, we will increase the number of samples by collecting more clinical specimens and group samples explicitly to ensure the accuracy of the research results.

In conclusion, we verified an explicit correlation between the elafin expression level and the prognosis of OC patients. Elafin expression was upregulated in patients with unfavorable prognoses, so we constructed a nomogram model for forecasting. Moreover, elafin expression was positively correlated with B cell memory, T cell CD4 naive, macrophage M1, and neutrophils. The mentioned TICs were associated with an ideal immunotherapy response, corresponding with the analyses of pathway enrichment. Herein our findings indicated that elafin could be a solid marker for the prognosis and immunotherapy response for OC.

## Data availability statement

The original contributions presented in the study are included in the article/**Supplementary Material**. Further inquiries can be directed to the corresponding author.

## Author contributions

WL and GT conceived and designed this study. BX collected the relative data and WL analyzed it. WL wrote the manuscript. MW conducted the IHC experiments. MW, BX, WD, SP, FL, and YW revised the manuscript. WD, JR, and KL checked the figures and tables. MW supervised the whole study. All authors contributed to the article and approved the submitted version.
