# Supplementary material for: Elafin is related to immune infiltration and could predict the poor prognosis in ovarian cancer
Source: Front Endocrinol (Lausanne). 2023 Jan 19;14:1088944. doi: 10.3389/fendo.2023.1088944 (PMC9893492; doi:10.3389/fendo.2023.1088944)
Supplement: Supplementary file 1 [file DataSheet_1.docx]

Supplementary Material

# Supplementary Figures and Tables

## Supplementary Figures


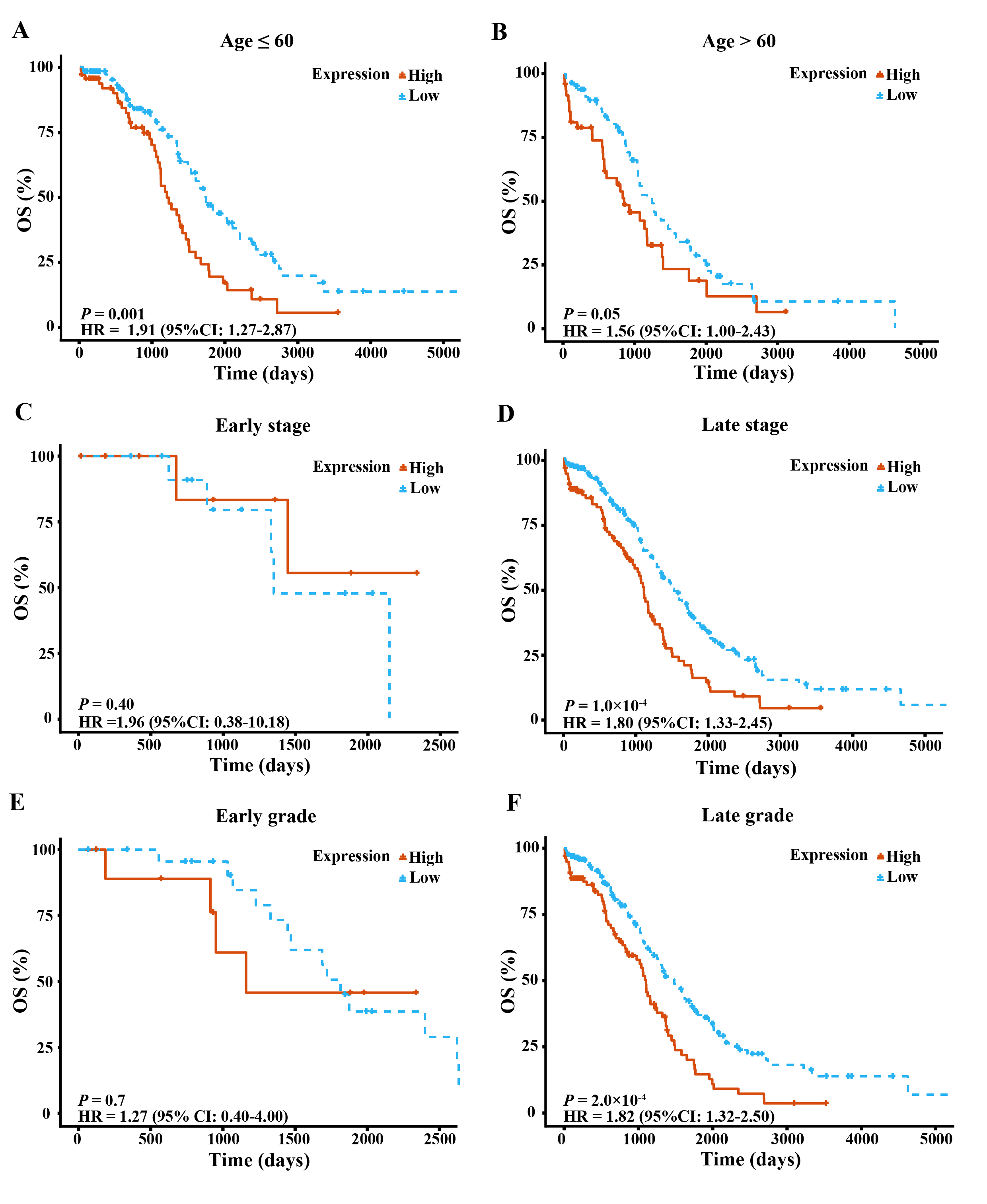


**Supplementary Figure. 1** KM survival curves of elafin with clinical characteristics in OC patients. **(a)** Age ≤ 60. **(b)** Age > 60. **(c)** Early grade. **(d)** Late grade. **(e)** Early stage. **(f)** Late stage.

**
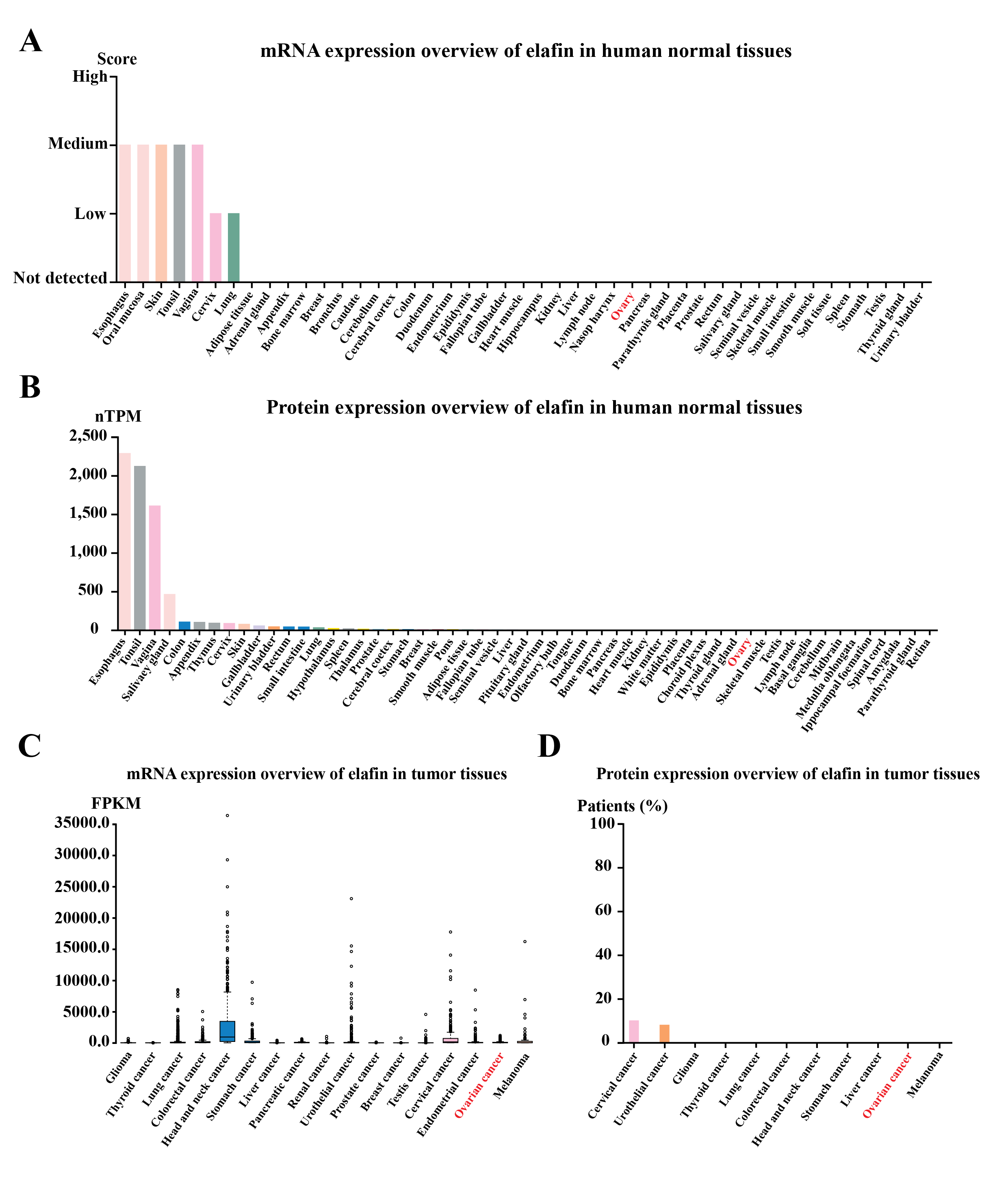
**

**Supplementary Figure. 2** The protein mRNA expression of elafin in normal and tumor tissues. **(a)** mRNA expression in human normal tissues. **(b)** Protein expression in human normal tissues. **(c)** mRNA expression in tumor tissues. **(d)** Protein expression in tumor tissues.

**
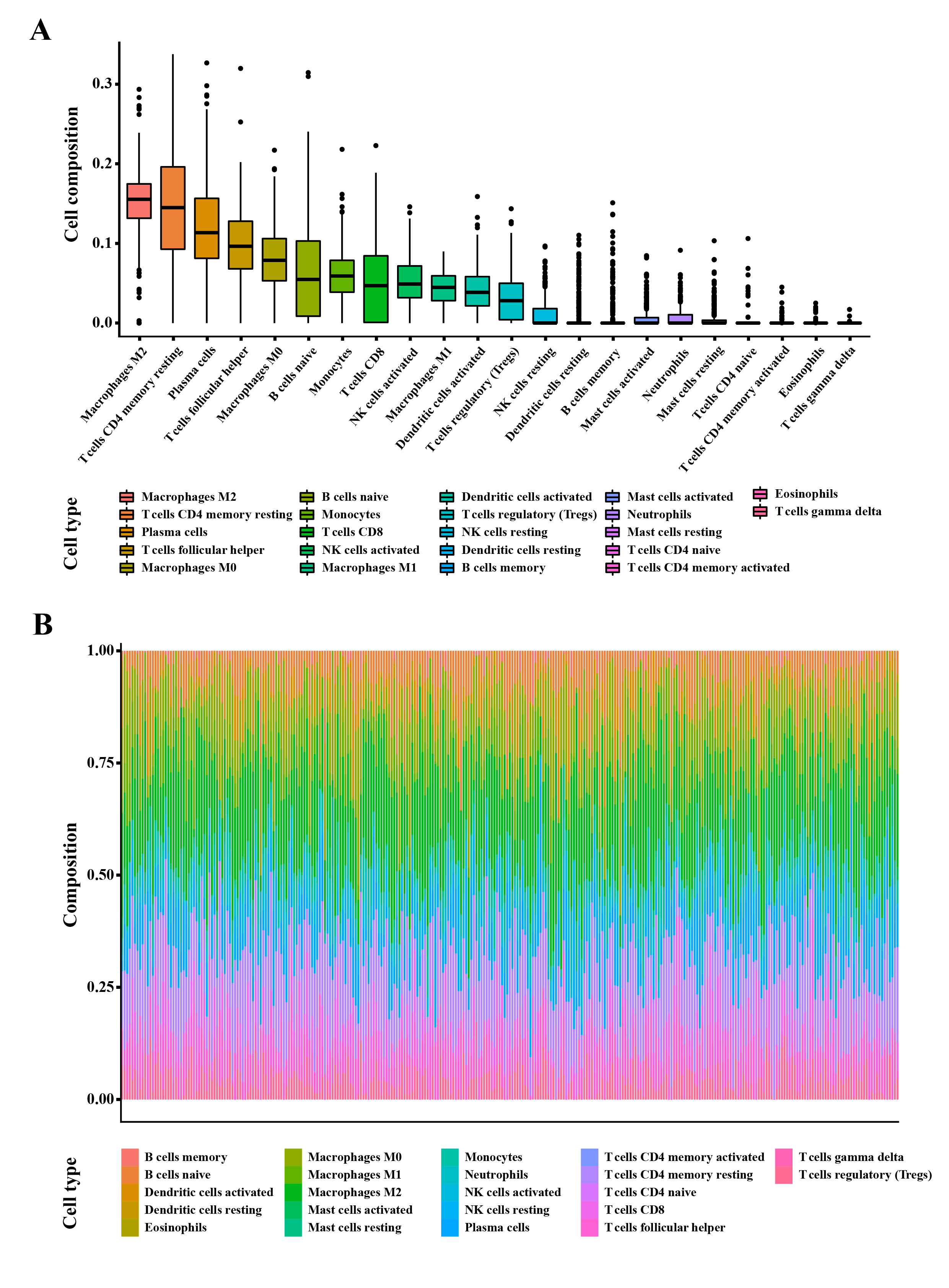
**

**Supplementary Figure. 3** Tumor immune cell infiltration in TCGA database. **(a)** The box plot shows the total composition of 22 types of TICs in descending order. **(b)** The stacked bar chart displays the proportion of 22 types of TICs in each sample


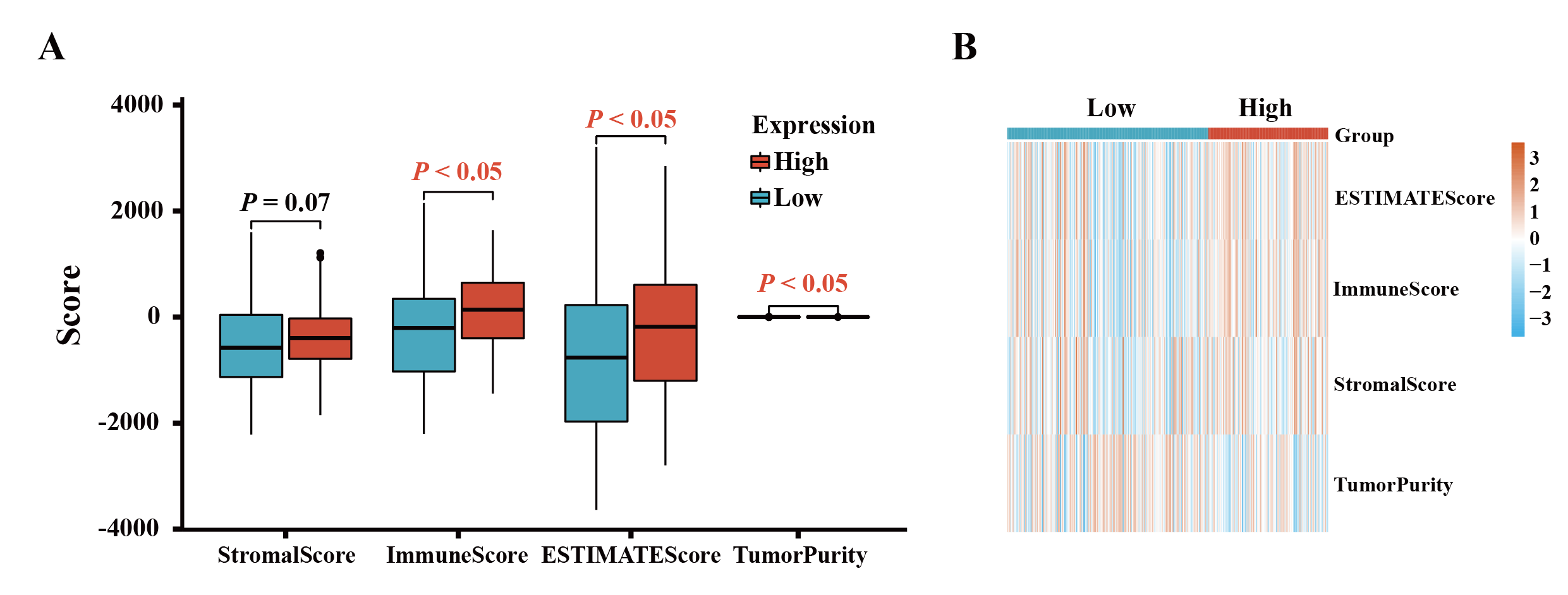


**Supplementary Figure. 4** The box plot **(a)** and heatmap **(b)** displays different ESTIMATE results in high- and low-expression groups.

## Supplementary Tables

**Supplementary Table 1. Clinic data of ovarian cancer samples from tissue microarray.**

|  | **Elafin protein expression (IHC score)** | | | | | | | | | |
| --- | --- | --- | --- | --- | --- | --- | --- | --- | --- | --- |
|  | **Tumor tissues (n = 42)** | | | | | **Adjacent tissues (n = 42)** | | | | |
|  | **Low** | **High** | ***P*-value** |  | | **Low** | | **High** | | ***P*-value** |
| **Total** | 32 (76%) | 10 (24%) | - | | | 12 (29%) | | 30 (71%) | | - |
| **Age** |  |  |  | | |  | |  | |  |
| ≤ 60 | 22 (69%) | 8 (80%) | 0.190 | | | 9 (75%) | | 21 (70%) | | 0.520 |
| > 60 | 10 (31%) | 2 (20%) |  | | 3 (25%) | | 9 (30%) | |  | |
| **TNM stage** |  |  |  | |  | |  | |  | |
| I/II | 12 (38%) | 4 (40%) | **0.045** | | 8 (67%) | | 8 (27%) | | 0.140 | |

**Continued**

| III/IV | 20 (62%) | 6 (60%) |  | 4 (33%) | 22 (73%) |  |
| --- | --- | --- | --- | --- | --- | --- |
| **pT status** |  |  |  |  |  |  |
| T1/T2 | 14 (44%) | 4 (40%) | 0.930 | 8 (67%) | 10 (33%) | **0.038** |
| T3 | 18 (56%) | 6 (60%) |  | 4 (33%) | 20 (67%) |  |
| **pN status** |  |  |  |  |  |  |
| N0 | 22 (69%) | 7 (70%) | 0.610 | 10 (83%) | 19 (63%) | 0.960 |
| N1 | 10 (31%) | 3 (30%) |  | 2 (17%) | 11 (37%) |  |
| **pM status** |  |  |  |  |  |  |
| M0 | 32 (100%) | 6 (60%) | **0.022** | 12 (100%) | 25 (83%) | **0.041** |
| M1 | 0 (0%) | 4 (40%) |  | 0 (0%) | 5 (17%) |  |
| **Histological subtype** | | | | | | |
| Serous | 28 (88%) | 5 (50%) | - | 8 (67%) | 24 (80%) | - |
| Endometrioid | 2 (6%) | 5 (50%) |  | 4 (33%) | 4 (14%) |  |
| Clear-cell carcinoma | 1 (3%) | 0 (0%) |  | 0 (0%) | 1 (3%) |  |
| Endometrioid and serous | 1 (3%) | 0 (0%) |  | 0 (0%) | 1 (3%) |  |
| **Recurrence of state** | | | | | | |
| Recurrence | 11 (%) | 5 (%) | 0.067 | 7 (58%) | 9 (30%) | **0.026** |
| No recurrence | 21 (%) | 5 (%) |  | 5 (42%) | 21 (70%) |  |

**Supplementary Table 2. Elafin-related drug resistance in OC predicted by GSCA.**

| **Drug** | **Correlation** | **FDR** | **Type** |
| --- | --- | --- | --- |
| Afatinib | -0.308 | 1.233E-20 | targeted therapy |
| Gefitinib | -0.283 | 3.628E-15 | targeted therapy |
| Cetuximab | -0.242 | 3.989E-11 | targeted therapy |
| Erlotinib | -0.378 | 5.454E-11 | targeted therapy |
| Lapatinib | -0.310 | 2.081E-08 | targeted therapy |
| Tubastatin A | 0.150 | 1.681E-05 | targeted therapy |
| PI-103 | 0.142 | 5.936E-05 | targeted therapy |
| XAV939 | -0.139 | 1.821E-04 | targeted therapy |
| Methotrexate | 0.135 | 2.017E-04 | chemotherapy |
| CX-5461 | 0.128 | 3.144E-04 | chemotherapy/immune-related targeted therapy |
| RO-3306 | -0.143 | 3.306E-04 | immune-related targeted therapy |
| Nutlin-3a (-) | 0.145 | 3.768E-04 | targeted therapy |
| BHG712 | 0.126 | 3.828E-04 | targeted therapy |
| TG101348 | 0.123 | 4.746E-04 | targeted therapy |
| Navitoclax | 0.125 | 6.359E-04 | targeted therapy |
| Trametinib | -0.122 | 7.023E-04 | targeted therapy |
| Docetaxel | -0.122 | 9.519E-04 | chemotherapy |
| PD-0325901 | -0.125 | 1.068E-03 | targeted therapy |
| Vorinostat | 0.119 | 1.086E-03 | targeted therapy |
| IPA-3 | 0.122 | 1.218E-03 | targeted therapy |

**Continued**

| TW 37 | 0.126 | 1.311E-03 | targeted therapy |
| --- | --- | --- | --- |
| AZD8055 | 0.119 | 1.875E-03 | targeted therapy |
| Camptothecin | 0.114 | 4.335E-03 | chemotherapy |
| Talazoparib | 0.106 | 9.018E-03 | immune-related targeted therapy |
| Foretinib | 0.098 | 1.112E-02 | targeted therapy |
| Bicalutamide | -0.103 | 1.195E-02 | chemotherapy |
| PAC-1 | 0.094 | 1.854E-02 | targeted therapy |
| 17-AAG | -0.088 | 1.862E-02 | targeted therapy |
| SN-38 | 0.094 | 1.948E-02 | chemotherapy |
| Dabrafenib | 0.093 | 2.090E-02 | targeted therapy |
| Etoposide | 0.096 | 2.478E-02 | chemotherapy |
| Belinostat | 0.082 | 2.649E-02 | chemotherapy |
| Gemcitabine | 0.095 | 3.090E-02 | chemotherapy |
| Pazopanib | 0.097 | 3.987E-02 | chemotherapy |
| LAQ824 | 0.079 | 4.210E-02 | chemotherapy |

| **Gene sets** | **Name** | **ES** | **NES** | **NOM *P*-value** | **FDR *Q*-value** | **Size** |
| --- | --- | --- | --- | --- | --- | --- |
| HALLMARK | Allograft rejection | 0.762 | 3.142 | 0 | 0 | 195 |
| HALLMARK | Inflammatory response | 0.712 | 2.978 | 0 | 0 | 197 |
| HALLMARK | Interferon-γ response | 0.713 | 2.927 | 0 | 0 | 196 |
| HALLMARK | IL6 JAK STAT3 signaling | 0.741 | 2.734 | 0 | 0 | 87 |
| HALLMARK | TNFA signaling via NFKB | 0.658 | 2.676 | 0 | 0 | 197 |
| HALLMARK | Interferon-α response | 0.710 | 2.547 | 0 | 0 | 92 |
| HALLMARK | KRAS signaling up | 0.578 | 2.381 | 0 | 0 | 193 |
| HALLMARK | Complement | 0.564 | 2.320 | 0 | 0 | 195 |
| HALLMARK | IL2 STAT5 signaling | 0.540 | 2.240 | 0 | 0 | 194 |
| HALLMARK | Apoptosis | 0.522 | 2.068 | 0 | 0 | 158 |
| HALLMARK | Coagulation | 0.502 | 2.039 | 0 | 0 | 136 |
| HALLMARK | Epithelial mesenchymal transition | 0.476 | 1.938 | 0 | 0 | 194 |

**Supplementary Table 3. Pathways significantly enriched in high-expression elafin group from GSEA analyses.**

**Continued**

| HALLMARK | Cholesterol homeostasis | 0.468 | 1.675 | 0.022 | 0.002 | 72 |
| --- | --- | --- | --- | --- | --- | --- |
| HALLMARK | Reactive oxygen species pathway | 0.500 | 1.665 | 0 | 0.002 | 45 |
| HALLMARK | P53 pathway | 0.392 | 1.620 | 0 | 0.005 | 190 |
| HALLMARK | UV response up | 0.383 | 1.617 | 0 | 0.005 | 152 |
| HALLMARK | Xenobiotic metabolism | 0.394 | 1.616 | 0 | 0.005 | 196 |
| HALLMARK | Oxidative phosphorylation | 0.386 | 1.598 | 0 | 0.006 | 182 |
| HALLMARK | Apical junction | 0.366 | 1.541 | 0 | 0.010 | 193 |
| HALLMARK | Hypoxia | 0.369 | 1.474 | 0 | 0.020 | 190 |
| HALLMARK | Estrogen response late | 0.360 | 1.452 | 0 | 0.021 | 195 |
| HALLMARK | Glycolysis | 0.326 | 1.390 | 0.023 | 0.036 | 195 |
| HALLMARK | Adipogenesis | 0.338 | 1.361 | 0 | 0.047 | 189 |
| HALLMARK | KRAS signaling down | 0.293 | 1.250 | 0.043 | 0.129 | 188 |
| HALLMARK | Estrogen response early | 0.293 | 1.189 | 0.022 | 0.186 | 192 |
| KEGG | Lysosome | 0.491 | 2.192 | 0 | 0.005 | 119 |

**Continued**

| KEGG | Nod-like receptor signaling pathway | 0.735 | 1.964 | 0.002 | 0.068 | 60 |
| --- | --- | --- | --- | --- | --- | --- |
| KEGG | Cytosolic DNA sensing pathway | 0.675 | 1.921 | 0 | 0.078 | 54 |
| KEGG | Toll-like receptor signaling pathway | 0.652 | 1.908 | 0 | 0.066 | 101 |
| KEGG | Apoptosis | 0.530 | 1.874 | 0.002 | 0.072 | 86 |
| KEGG | Leishmania infection | 0.756 | 1.873 | 0.002 | 0.061 | 70 |
| KEGG | Systemic lupus erythematosus | 0.777 | 1.853 | 0.002 | 0.061 | 54 |
| KEGG | Antigen processing and presentation | 0.712 | 1.834 | 0.004 | 0.064 | 80 |
| KEGG | B cell receptor signaling pathway | 0.572 | 1.818 | 0.008 | 0.064 | 74 |
| KEGG | Chemokine signaling pathway | 0.628 | 1.817 | 0 | 0.059 | 185 |
| KEGG | Intestinal immune network for iga production | 0.796 | 1.797 | 0.002 | 0.062 | 46 |
| KEGG | Cytokine cytokine receptor interaction | 0.671 | 1.773 | 0 | 0.068 | 258 |
| KEGG | Amino sugar and nucleotide sugar metabolism | 0.481 | 1.766 | 0.013 | 0.067 | 42 |
| KEGG | Autoimmune thyroid disease | 0.754 | 1.766 | 0.002 | 0.062 | 50 |
| KEGG | Snare interactions in vesicular transport | 0.412 | 1.742 | 0.015 | 0.073 | 38 |

**Continued**

| KEGG | T cell receptor signaling pathway | 0.586 | 1.730 | 0.025 | 0.074 | 106 |
| --- | --- | --- | --- | --- | --- | --- |
| KEGG | RIG I like receptor signaling pathway | 0.501 | 1.709 | 0.008 | 0.081 | 68 |
| KEGG | Asthma | 0.807 | 1.709 | 0.002 | 0.077 | 28 |
| KEGG | Type Ⅰ diabetes mellitus | 0.784 | 1.708 | 0.004 | 0.073 | 41 |
| KEGG | Allograft rejection | 0.835 | 1.695 | 0.004 | 0.077 | 35 |
| KEGG | Primary immunodeficiency | 0.766 | 1.689 | 0.008 | 0.078 | 35 |
| KEGG | Hematopoietic cell lineage | 0.692 | 1.686 | 0.006 | 0.076 | 84 |
| KEGG | Graft versus host disease | 0.852 | 1.669 | 0 | 0.083 | 37 |
| KEGG | Cell adhesion molecules cams | 0.599 | 1.643 | 0.017 | 0.096 | 128 |
| KEGG | Viral myocarditis | 0.569 | 1.640 | 0.024 | 0.093 | 68 |
| KEGG | Natural killer cell mediated cytotoxicity | 0.593 | 1.638 | 0.031 | 0.092 | 131 |
| KEGG | JAK STAT signaling pathway | 0.530 | 1.637 | 0.006 | 0.088 | 151 |
| KEGG | Leukocyte transendothelial migration | 0.485 | 1.603 | 0.025 | 0.105 | 114 |
| KEGG | Epithelial cell signaling in helicobacter pylori infection | 0.415 | 1.581 | 0.037 | 0.112 | 67 |

**Continued**

| KEGG | Endocytosis | 0.338 | 1.507 | 0.049 | 0.167 | 175 |
| --- | --- | --- | --- | --- | --- | --- |
| KEGG | Complement and coagulation cascades | 0.540 | 1.432 | 0.043 | 0.219 | 68 |
